# Evaluation of a pantoprazole and 4‐desmethylpantoprazole‐sulfate metabolic ratio as a novel CYP2C19 phenotyping method

**DOI:** 10.1002/bcp.70631

**Published:** 2026-05-31

**Authors:** Julian Peter Müller, Vladimir N. Belov, Sabrina Yamoune, Bob Miller, Mohammed Hasoumi, Jolanta Tupiec, Thea Laurentius, L. Cornelius Bollheimer, Julia C. Stingl, Katja S. Just

**Affiliations:** ^1^ Institute of Clinical Pharmacology University Hospital RWTH Aachen Aachen North Rhine‐Westphalia Germany; ^2^ Department of NanoBiophotonics Max Planck Institute for Multidisciplinary Sciences (MPI‐NAT) Göttingen Lower Saxony Germany; ^3^ Department of Geriatric Medicine University Hospital RWTH Aachen Aachen North Rhine‐Westphalia Germany; ^4^ Department of Geriatrics Carl von Ossietzky University of Oldenburg Oldenburg Lower Saxony Germany; ^5^ Department of Clinical Pharmacology and Pharmacoepidemiology University Hospital Heidelberg Heidelberg Baden‐Württemberg Germany

**Keywords:** CYP2C19, cytochrome P450, drug–drug interactions, personalized medicine, pharmacogenetics, pharmacokinetics

## Abstract

**Aims:**

CYP2C19 is one of the most important pharmacogenes, and its activity is highly variable due to factors such as genetics or drug–drug interactions. Due to the lack of an appropriate endogenous CYP2C19 biomarker, surrogate methods to assess its activity are warranted. The aim of this work was to validate pantoprazole as a CYP2C19 phenotyping drug.

**Methods:**

We performed a single‐dose pharmacokinetic study with 20 healthy participants in a randomized cross‐over design. Participants received 10 mg omeprazole and 20 mg pantoprazole on two different study days with a washout phase of one week. Blood sampling was performed at 14 time points spanning 8 h after drug administration. Drug and metabolite plasma concentrations were assessed by LC–MS/MS. Metabolic ratios (MR) of metabolite/substrate were formed using the area under the curve from 0 to 8 h (AUC_0‐8h_) and single plasma concentrations.

**Results:**

We demonstrate very strong correlations (Spearman ρ = 0.84) of AUC_0‐8h_ MR 4‐desmethylpantoprazole‐sulfate/pantoprazole to the validated CYP2C19 probe AUC_0‐8h_ MR 5‐hydroxyomeprazole/omeprazole (Spearman ρ = 0.84). Single time point pantoprazole MRs measured in plasma between 4 and 8 h after pantoprazole administration were sufficient for accurate CYP2C19 phenotyping by demonstrating significant and robust correlations to the AUC_0‐8h_ MR 5‐hydroxyomeprazole/omeprazole with Spearman ρ ≥ 0.8.

**Conclusions:**

In contrast to omeprazole, pharmacokinetics of pantoprazole are described to be stable during repeated dosing, which may allow phenotyping of CYP2C19 with patients using pantoprazole MRs in situations where pantoprazole is part of the prescribed medication.

**What is already known about this subject**

- Individual CYP2C19 activity is highly variable, but no endogenous biomarker is available
- The kinetics of the CYP2C19 probe drug omeprazole are not stable during repeated dosing due to CYP2C19 inhibition, but pantoprazole has less or no CYP2C19 auto‐inhibition and may be used as CYP2C19 activity marker during repeated dosing in clinical settings

**What this study adds:**

- The metabolic ratio (MR) 4‐desmethylpantoprazole‐sulfate/pantoprazole shows a strong correlation to the MR 5‐hydroxyomeprazole/omeprazole
- Using the pantoprazole MR, single plasma samples taken between 4 and 8 h after pantoprazole intake may allow accurate depiction of the CYP2C19 phenotype
- CYP2C19 phenotyping may be performed with patients currently on pantoprazole medication

## 1 INTRODUCTION

CYP2C19 is one of the most important pharmacogenes and is involved in the metabolism of about 10% of all marketed drugs.^1^, ^2^ Among its substrates are antidepressants such as citalopram and sertraline, the antiplatelet drug clopidogrel and proton pump inhibitors (PPI) such as omeprazole, pantoprazole and lansoprazole.^3^ CYP2C19 is genetically highly polymorphic, but the variability in CYP2C19 enzyme activity cannot be fully explained by the genotype,^1^ as shown in a study with the antifungal voriconazole, in which about 49% of its pharmacokinetic variance could be explained by CYP2C19 polymorphisms.^4^ Next to genetic variability, CYP2C19 activity can also be influenced by non‐genetic factors such as inflammation,^5^, ^6^ and enzyme induction or inhibition by drug–drug interactions, which may lead to phenoconversion.^2^ Phenoconversion is defined as a genotype–phenotype mismatch, precisely the difference between the genotype‐predicted phenotype and the real measurable phenotype.^2^

To further study CYP2C19 variability and phenoconversion in patient cohorts, a biomarker to measure enzyme activity in vivo would be desirable. While there are reports hinting at arachidonic acid and epoxyeicosatrienoic acids (EETs) as potential endogenous markers, the information is scarce.^7^, ^8^, ^9^ Due to the lack of an accurate endogenous CYP2C19 biomarker, and the urgent need to study factors of phenoconversion in patients, e.g., in a polypharmacy setting, CYP2C19 phenotyping may be performed using already prescribed medication.^10^ Suitable and widely prescribed candidates for such an approach may be PPIs, such as omeprazole or pantoprazole.^11^ Both are major CYP2C19 substrates with minor involvement of CYP3A4, in particular for omeprazole.^12^, ^13^

In single‐dose studies, phenotyping of CYP2C19 is commonly performed using omeprazole as a probe drug,^14^, ^15^ but with multiple dosing, as in clinical prescriptions, omeprazole may lead to inaccurate phenotyping results due to its time‐dependent inhibition of CYP2C19.^16^, ^17^, ^18^, ^19^ CYP2C19 inhibition by esomeprazole has been shown to be long‐lasting and may last up to 3–4 days,^18^ correspondingly the area under the curve (AUC) of esomeprazole increased with multiple dosing.^20^ In contrast, less or no CYP2C19 inhibition by pantoprazole was reported,^21^, ^22^, ^23^ and the pantoprazole AUC remains stable during repeated dosing.^24^ Therefore, pantoprazole may be a much better candidate for CYP2C19 phenotyping from the prescribed medication and during repeated dosing than the widely established probe drug omeprazole. Literature reports are promising, as pantoprazole was previously studied as a CYP2C19 activity marker, demonstrating that its pharmacokinetics are influenced by the CYP2C19 genotype,^25^, ^26^ and a pantoprazole breath test was assessed for CYP2C19 activity in children and healthy volunteers.^27^, ^28^, ^29^

This study aims to test whether pantoprazole metabolic ratios could be used as a probe drug for CYP2C19 activity by assessing the correlation with the validated CYP2C19 probe drug omeprazole.

## 2 METHODS

### 2.1 Study design

An open label, randomized, single‐dose pharmacokinetic study in healthy volunteers was conducted in cross‐over design. The study followed the Declaration of Helsinki. It was registered in the German Clinical Trials Register with the number DRKS00031441, and approved by the Independent Ethics Committee of the Faculty of Medicine of the RWTH Aachen University (approval number 23/087).

Inclusion criteria were healthy adults between 18 and 60 years, a body‐mass index (BMI) between 18 and 30 and written informed consent. Exclusion criteria included pregnancy and breastfeeding, hypersensitivity towards the administered drugs or one of its ingredients, regular intake of drugs (except antiallergic and antiasthmatic drugs and thyroid hormones), intake of proton‐pump‐inhibitors, alcohol or cannabis in the week prior the study.

When initial inclusion criteria were met, eligible volunteers were invited for a first study visit a minimum of 24 h or maximum of 14 days prior the first study day, on which they were comprehensively informed about the study. The volunteers were thoroughly examined for inclusion and exclusion criteria including medical history, physical examination, vital parameters, urine analysis, clinical chemistry and body temperature. For CYP2C19 genotyping, blood samples (7 mL each) were drawn into ethylenediaminetetraacetic acid (EDTA) containing tubes, which were stored at −32 °C until analysis.

The sample size was estimated using previous data on the intra‐individual coefficients of variations (CV) of the AUCs of omeprazole and pantoprazole, which are estimated to be between 5 to 30%.^30^, ^31^ Due to the controlled setting of our study design, that study participants presented fasted in the morning, and the study days were temporally close to each other (one week), we assumed a reduction in variability to a CV of 20 to 25%. Based on these assumptions we estimated the sample size for a study in cross‐over design. The probability of α error was set to 5% and the power (1‐β) to 80%. Using an iterative calculation and recommendations led to a minimum sample size of 20.^32^

20 healthy participants were enrolled and on the first study day were randomly allocated to pantoprazole or omeprazole using the tool https://www.randomizer.at and received either a single oral dose of 10 mg omeprazole or 20 mg pantoprazole, and after a washout phase of 1 week received the respective other drug at the second study day. On the study days, a brief health check was performed, and if no health‐related concerns existed, omeprazole or pantoprazole were administered. The subjects presented to the study facilities fasted in the morning. Three hours after drug intake the subjects received a meal on both study days. The blood sampling was performed over 14 time points at 0, 0.5, 1, 1.5, 2, 2.5, 3, 3.5, 4, 4.5, 5, 6, 7 and 8 h after pantoprazole or omeprazole intake using a peripheral venous catheter. Venous blood samples (7 mL each) were drawn into EDTA‐containing tubes. Blood samples were centrifuged immediately after blood sampling for 10 min at 2000*g* to generate blood plasma. Blood plasma was aliquoted and stored at −32 °C until analysis.

### 2.2 Materials

TaqMan assays for CYP2C19 genotyping (all ThermoFisher Scientific): CYP2C19*2 (rs4244285, C__25986767_70), CYP2C19*3 (rs4986893, C__27861809_10) and CYP2C19*17 (rs12248560, C____469857_10).*rac‐*Pantoprazole (Cay21345–1, Biomol), *rac‐*pantoprazole‐d6 (Cay28483–1, Biomol), *rac‐*4‐desmethyl pantoprazole (B3060936, BenchChem), *rac‐*omeprazole (HY‐B0113, MedChemExpress), 5‐hydroxyomeprazole (Cay10009028–500, Cayman Chemical), *rac‐*omeprazole‐d3 (CMS‐CS‐4014‐1MG, Biozol), *rac‐*5‐hydroxyomeprazole‐d3 sodium salt (TOR‐H948864‐1MG, Biozol), methanol (HPLC gradient grade, 8402.2500, J.T. Baker), water (LiChrosolv LC–MS grade, MC1153332500, Merck), formic acid (HiPersolv CHROMANORM LC–MS grade, 84.865.180, VWR), bovine serum (2203–010, Acila), dimethyl sulfoxide (DMSO; A994.2, ROTH).

### 2.3 CYP2C19 genotyping

CYP2C19 genotyping was performed using a QuantStudio 6 Pro qPCR‐Machine (Applied Biosystems) and TaqMan assays according to the manufacturer's instructions. The study participants were genotyped for the CYP2C19 variants *2 (rs4244285), *3 (rs4986893) and *17 (rs12248560). In sum these variants and the wild type (*1) represent 98.8% of CYP2C19 genetic variability in Europeans.^33^ Genotype‐predicted phenotypes were assigned from the diplotypes according to CPIC guidelines.^33^, ^34^

### 2.4 Synthesis of 4‐desmethylpantoprazole‐sulfate and 4‐desmethylpantoprazole‐sulfate‐d3

The main pantoprazole metabolite in human plasma 4‐desmethylpantoprazole‐4‐O‐sulfate^35^ and its deuterated analog with a trideuteriomethoxy group attached to C‐3 in the pyridine ring, were synthesized using a novel synthetic methodology. This methodology is applicable for the preparation of various “metabolic” O‐sulphates from drugs and drug candidates containing 4‐methoxypyridine or 2‐methoxypyridine residues, e.g., omeprazole, tenatoprazole and beyond. The detailed description, including the reaction schemes, is given in the supplemental information (Figure S1 and S2). Briefly, 2‐chloromethyl‐3,4‐dimethoxypyridine and its deuterium labelled analogue (2‐chloromethyl‐3‐(D_3_)‐methoxy‐4‐methoxypyridine were used as a starting material in an S‐alkylation reaction with 5‐(difluoromethoxy)‐2‐mercapto‐1*H*‐benzimidazol (compound 3, Figure S1) to prepare the pantoprazole molecule with a non‐oxidized S‐atom (see compound 4, Figure S1).^36^ Selective demethylation at C‐4 of the pyridine ring was performed by heating with anhydrous sodium sulphide in N‐methylpyrrolidone.^36^ Protection of benzimidazole nitrogen was performed using 2,2,2‐trichloroethoxycarbonyl (Troc) during the sulfation reaction with SO_3_ pyridine complex in DMF – pyridine solution.^37^ The Troc group was hydrolyzed in aqueous trimethylamine bicarbonate buffer at pH 9, and the resulting product (see compound 8, Figure S1) was isolated as triethylammonium salt by preparative HPLC. Finally, the sulphur atom was oxidized using sodium hypochlorite.^38^ Excessive sodium hypochlorite was destroyed by aqueous sodium bisulphite, and the reaction mixture was lyophilized. The final products 4‐desmethylpantoprazole‐sulfate and 4‐desmethylpantoprazole‐sulfate‐d3 were isolated by preparative HPLC. The product identities were verified by NMR and LC–MS mass spectra (Figure S3‐S7).

### 2.5 Determination of probe drugs and metabolites

Probe drugs and metabolite concentrations were determined using a liquid‐chromatography tandem mass spectrometer (LC–MS/MS) system consisting of an Agilent 1290 Infinity II UHPLC coupled to a Sciex QTRAP6500 + triple quadrupole mass spectrometer.

### 2.6 Measurement of omeprazole and 5‐hydroxyomeprazole

Plasma concentrations of omeprazole and 5‐hydroxyomeprazole were assessed using a previously published LC–MS/MS method,^39^ with slight modification. The total chromatographic method length was increased from 8.5 to 8.8 min. Briefly, 20 μl plasma were protein precipitated with 80 μl methanol including internal standards omeprazole‐d3 and 5‐hydroxyomeprazole‐d3. Samples were vortexed for 10 s and centrifuged for 20 min at 17.000 x *g* and 4 °C. A total of 40 μl supernatant were diluted with 40 μl LC grade water, vortexed briefly and centrifuged again for 5 min at 17.000 x *g* and 4 °C. A total of 50 μl of the resulting supernatant were transferred to a LC vial and 5 μl were injected onto a Poroshell 120 EC‐C18 column (1.9 μm, 2.1 x 50 mm, 699 675–902, Agilent Technologies) for analysis. The accuracy and precision over all measured study batches are shown in Table S1.

### 2.7 Measurement of pantoprazole and 4‐desmethylpantoprazole‐sulfate

Plasma concentrations of pantoprazole and 4‐desmethylpantoprazole‐sulfate were assessed using a novel LC–MS/MS method. A total of 20 μl plasma were protein precipitated with 380 μl methanol including the internal standards pantoprazole‐d6 (6.25 ng ml^−1^) and 4‐desmethylpantoprazole‐sulfate‐d3 (1.25 ng ml^−1^). The samples were vortexed for 10 s and centrifuged for 20 min at 4 °C and 17.000 x *g*. A total of100 μl of the supernatant were transferred to a LC vial and 5 μl were injected for analysis.

Samples were separated on a Poroshell 120 PFP column (2.6 μm, 2.1 x 50 mm, 699 775–408, Agilent Technologies) with A: 0.1% formic acid (v/v) in water and B: methanol with a flow rate of 0.6 mL/min and a total method length of 6 min. The gradient representing gradual increases over time was as follows: 2% B at 0 min, 65% B at 3.3 min, 100% B at 3.4 min, 100% B at 4.4 min, 2% B at 4.5 min, 2% B at 6 min.

Pantoprazole and 4‐desmethylpantoprazole‐sulfate were quantified using selected multiple reaction monitoring (sMRM) in positive and negative ion mode, respectively. Mass transitions for pantoprazole were 384 ➔ 200 *m/z* (quantifier), 384 ➔ 138 *m/z* (qualifier) and 390 ➔ 141 *m/z* (internal standard). The mass transitions for 4‐desmethylpantoprazole‐sulfate were 448 ➔ 350 *m/z* (quantifier), 448 ➔ 230 *m/z* (qualifier) and 453 ➔ 355 *m/z* (internal standard). For the detection of the internal standard 4‐desmethylpantoprazole‐sulfate‐d3, the +2 isotope was used to prevent interference from the analyte signal of 4‐desmethylpantoprazole‐sulfate.

Calibration curves for pantoprazole and 4‐desmethylpantoprazole‐sulfate were constructed in bovine serum with a concentration range from 3 to 2400 ng ml^−1^ for pantoprazole and 4 to 1600 ng ml^−1^ for 4‐desmethylpantoprazole‐sulfate with a minimum of 7 non‐zero standards and 5 quality controls. The resulting calibration curves were weighted with 1/x^2^. The average accuracy and precision over all measured study batches was within ±15% and below 15% CV (Table S2).

### 2.8 Validation of pantoprazole and 4‐desmethylpantoprazole‐sulfate method

The method for pantoprazole and 4‐desmethylpantoprazole‐sulfate measurement was validated for intra‐ and interday accuracy and precision, 24 h auto sampler stability at 10 °C, 24 h benchtop stability at room‐temperature, 3x freeze–thaw stability, matrix effect and dilution integrity. The method validation data are shown in Tables S3‐S6.

### 2.9 Calculation and statistics

The AUC from 0 to 8 h (AUC_0‐8h_) for drugs and metabolites was calculated using the lin‐lin method with Microsoft Excel 16. The AUC_0‐8h_ metabolic ratios (MR) were calculated as follows: AUC_0‐8h_ MR = AUC_0‐8h, metabolite_/AUC_0‐8h, substrate_. For single time‐point MR the calculation was: MR = C_metabolite_/C_substrate_. The correlations between the MR of 5‐hydroxyomeprazole/omeprazole and 4‐desmethylpantoprazole‐sulfate/pantoprazole were assessed by using non‐parametric Spearman correlation tests. The discrimination of CYP2C19 genotype‐predicted intermediate and rapid metabolizers from CYP2C19 normal metabolizers by pantoprazole or omeprazole MR are shown as boxplots with whiskers depicting the min and max values, lower and upper quartile and the median. Statistical significance was assessed using non‐parametric Kruskal‐Wallis test with Dunn's post hoc test. A p‐value < 0.05 was considered significant. Statistical analyses were performed using GraphPad Prism 10.

### 2.10 Nomenclature of targets and ligands

Key protein targets and ligands in this article are hyperlinked to corresponding entries in https://www.guidetopharmacology.org, and are permanently archived in the Concise Guide to PHARMACOLOGY 2023/2024.^40^, ^41^

## 3 RESULTS

In the study cohort, 14 participants were male and 6 female. The median age (range) of the participants was 24 (22–27) years, their median BMI was 23.5 (19.7–28.1) kg/m^2^, and their estimated median glomerular filtration was 115.55 (97.6–130.2) ml/min/1.73 m^2^ (Table S7). The study participants were genotyped for CYP2C19 and 4 intermediate (IM, 20%), 11 normal (NM, 55%) and 5 rapid metabolizers (RM, 25%) were identified. No poor (PM) or ultrarapid metabolizers (UM) were detected in the study cohort (Table S8).

The major route of pantoprazole metabolism is demethylation by CYP2C19 at the 4‐position, followed by sulfotransferase‐mediated sulfation at the same position, leading to 4‐desmethylpantoprazole‐sulfate.^35^, ^42^ We initially aimed to determine 4‐desmethylpantoprazole to allow the calculation of metabolic ratios (MR), but observed a rapid degradation of this analyte with a half‐life of 67.5 min in a methanol/water mixture (Figure S8), likely due to the free hydroxyl‐group at position C‐4 of the pyridine ring, which is prone to oxidation (Figure S9). Instead, we focused on the more stable phase II metabolite 4‐desmethylpantoprazole‐sulfate as demonstrated by stability data obtained during analytical method validation (Table S4). The analytical standard 4‐desmethylpantoprazole‐sulfate and its trideuteriomethoxy analogue were synthesized using a novel synthesis route to prepare O‐sulphates from drugs and drug candidates containing 4‐methoxypyridine and 2‐methoxypyridine residues (Figure S1 and S2). The standards were used to develop and validate a novel LC–MS/MS method that allows the simultaneous quantification of pantoprazole and its metabolite 4‐desmethypantoprazole‐sulfate in plasma.

Plasma concentration time curves of pantoprazole, omeprazole and its metabolites yielded characteristic curves (Figure 1A and B). The t_max_ of pantoprazole and omeprazole were 2 and 1.5 h, and the C_max_ were 1058 ± 712 ng ml^−1^ and 136 ± 151 ng ml^−1^ for pantoprazole and omeprazole, respectively. The plasma concentrations from 0 to 8 h were used to calculate AUC_0‐8h_ of pantoprazole, omeprazole and their metabolites, and the respective AUC_0‐8h_ MR by dividing AUC_0‐8h, metabolite_ by AUC_0‐8h, substrate_.

**FIGURE 1:**
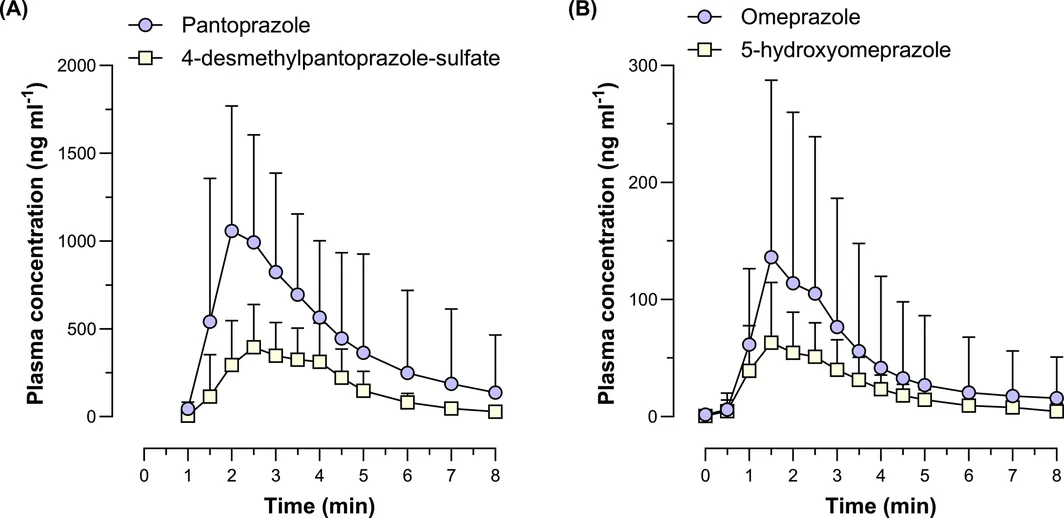
Plasma concentration time profile of pantoprazole and omeprazole and respective metabolites in 20 healthy volunteers after a single‐dose oral administration of 20 mg pantoprazole and 10 mg omeprazole. A: plasma concentration time curve of pantoprazole and 4‐desmethylpantoprazole‐sulfate. B: plasma concentration time curve of omeprazole and 5‐hydroxyomeprazole. Data are given as mean + standard deviation.

Analysis of the correlation between the ln AUC_0‐8h_ MR of 4‐desmethylpantoprazole‐sulfate/pantoprazole and the ln AUC0‐8h MR of 5‐hydroxyomeprazole/omeprazole revealed a significant and strong positive correlation with a Spearman ρ of 0.84 and a p‐value of <0.0001 (Figure 2A). In addition, the correlation of the pantoprazole AUC_0‐8h_ to the CYP2C19 phenotype, depicted by ln AUC_0‐8h_ MR of 5‐hydroxyomeprazole/omeprazole, was tested. It showed a significant negative correlation with a Spearman ρ of −0.76 with a p‐value of <0.0001 (Figure 2B).

**FIGURE 2:**
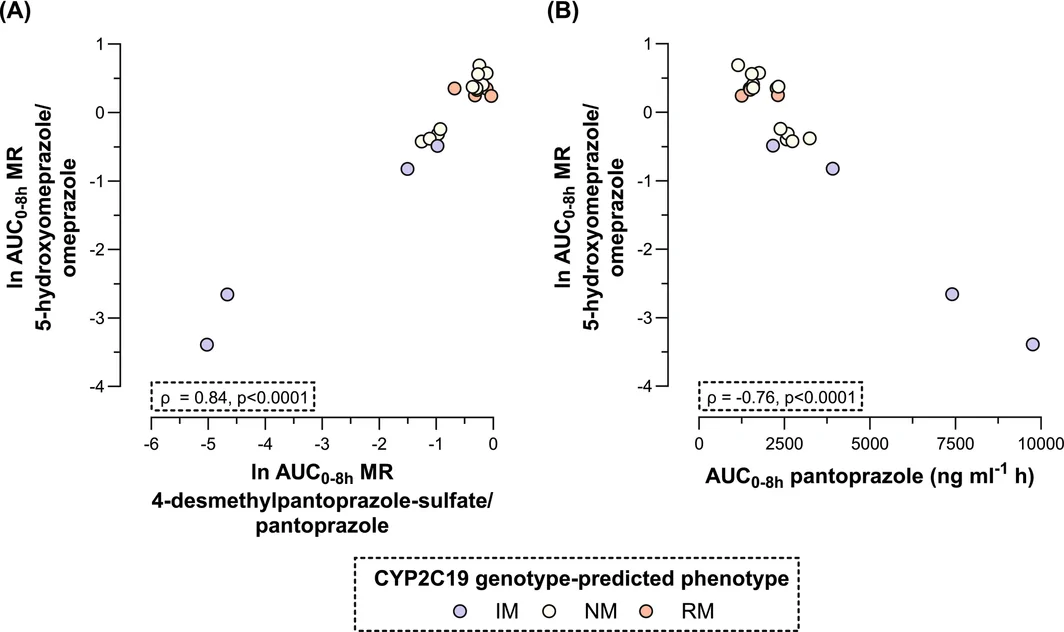
Spearman correlation of ln AUC_0‐8h_ MR omeprazole to ln AUC_0‐8h_ MR pantoprazole and the pantoprazole AUC_0‐8h_. A: correlation of the ln AUC_0‐8h_ MR 5‐hydroxyomeprazole/omeprazole to ln AUC_0‐8h_ MR 4‐desmethylpantoprazole‐sulfate/pantoprazole. B: correlation of ln AUC_0‐8h_ MR 5‐hydroxyomeprazole/omeprazole to AUC_0‐8h_ pantoprazole. Ln‐transformed data of AUC_0‐8h_ MR 5‐hydroxyomeprazole/omeprazole and AUC_0‐8h_ MR 4‐desmethylpantoprazole‐sulfate/pantoprazole are shown. Spearman ρ is given, and a p‐value < 0.05 was considered significant. AUC: area under the curve; MR: metabolic ratio; IM: intermediate metabolizer; NM: normal metabolizer; RM: rapid metabolizer.

The data are shown without ln‐transformation in Figure S10A‐B, highlighting potential non‐linear relationships. Correlation analyses were as well performed after exclusion of two outlying CYP2C19 IM subjects. These were still significant with ρ of 0.78, p‐value = 0.0001 and ρ of −0.67, p‐value = 0.0023 for AUC MR omeprazole *vs*. pantoprazole and AUC MR omeprazole *vs*. AUC pantoprazole, respectively (Figure S10C‐D).

The ability of the AUC_0‐8h_ MR omeprazole and pantoprazole to discriminate between the CYP2C19 genotype‐predicted phenotypes were assessed. Both AUC_0‐8h_ MR of omeprazole and pantoprazole were able to significantly discriminate IMs from NMs (Figure 3A and B). Neither AUC_0‐8h_ MR pantoprazole nor omeprazole were able to discriminate NMs from RMs (Figure 3A and B).

**FIGURE 3:**
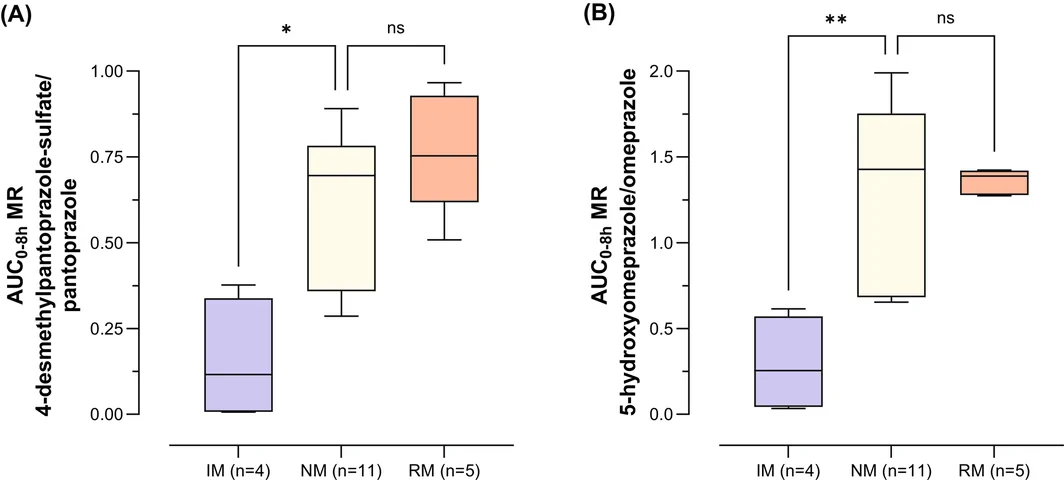
Relationship of CYP2C19 genotype‐predicted phenotype and the pantoprazole and omeprazole metabolic ratios (MR). A: ability of AUC_0‐8h_ MR 4‐desmethylpantoprazole‐sulfate/pantoprazole to discriminate between CYP2C19 phenotypes. B: ability of AUC_0‐8h_ MR 5‐hydroxyomeprazole/omeprazole to discriminate between CYP2C19 phenotypes. The data are shown as boxplots with whiskers depicting min and max values, lower and upper quartiles, and the median. Statistical significance was assessed using a non‐parametric Kruskal‐Wallis test with Dunn's post hoc test. P‐value < 0.05 was considered significant. * = p < 0.05, ** = p < 0.01; AUC: area under the curve; MR: metabolic ratio; IM: intermediate metabolizer; NM: normal metabolizer; RM: rapid metabolizer.

To facilitate the use of pantoprazole for CYP2C19 phenotyping from already prescribed medication in the clinic, the use of single plasma samples would be favourable. Hence, the single‐time‐point MR of 4‐desmethylpantoprazole‐sulfate/pantoprazole and the single plasma concentrations of pantoprazole were compared to the CYP2C19 phenotype depicted by the AUC_0‐8h_ MR 5‐hydroxyomeprazole/omeprazole. We observed significant and strong positive correlations at each of the single time‐point pantoprazole MR between 4 and 8 h after pantoprazole administration (Spearman ρ ≥ 0.8). The correlation to AUC_0‐8h_ MR omeprazole was consistently stronger using single time‐point pantoprazole MR compared to single plasma concentrations of pantoprazole (Spearman ρ between – 0.34 and −0.8) (Table 1).

**TABLE 1:** Correlation of single time point MR 4‐desmethylpantoprazole‐sulfate/pantoprazole and single time point pantoprazole plasma concentrations to the AUC_0‐8h_ MR 5‐hydroxyomeprazole/omeprazole. Rows depicting correlations with a Spearman ρ ≥ 0.8 and number of pairs ≥ 19 are written with bold text.

Sampling time (hours)	MR 4‐desmethylpantoprazole‐sulfate/pantoprazole				Pantoprazole concentration			
	Spearman ρ	p‐value	Significance	Number of pairs	Spearman ρ	p‐value	Significance	Number of pairs
0.0	n.d.	n.d.	n.d.	n.d.	n.d.	n.d.	n.d.	n.d.
0.5	n.d.	n.d.	n.d.	n.d.	n.d.	n.d.	n.d.	n.d.
1.0	n.d.	n.d.	n.d.	1	n.d.	n.d.	n.d.	2
1.5	0.86	0.0238	*	7	−0.10	0.8401	ns	8
2.0	0.63	0.0323	*	12	−0.16	0.604	ns	13
2.5	0.66	0.0128	*	14	−0.41	0.1334	ns	15
3.0	0.64	0.0093	**	16	−0.56	0.0216	*	17
3.5	0.62	0.006	**	18	−0.50	0.0344	*	18
4.0	**0.80**	**<0.0001**	****	**19**	−0.34	0.1517	ns	19
4.5	**0.85**	**<0.0001**	****	**19**	−0.51	0.0217	*	20
5.0	**0.88**	**<0.0001**	****	**20**	−0.62	0.0038	**	20
6.0	**0.90**	**<0.0001**	****	**20**	−0.72	0.0003	***	20
7.0	**0.91**	**<0.0001**	****	**20**	−0.76	0.0001	***	20
8.0	**0.91**	**<0.0001**	****	**20**	**−0.80**	**<0.0001**	****	**20**

n.d.: not determined, AUC: area under the curve, MR: metabolic ratio.

* p < 0.05.

** p < 0.01.

*** p < 0.001.

**** p < 0.0001.

## 4 DISCUSSION

With this study, our aim was to offer another probe drug potentially usable for CPY2C19 phenotyping in vivo. To the best of our knowledge, herein we demonstrate for the first time a very strong and robust correlation between the AUC_0‐8h_ MR of the validated CYP2C19 probe drug omeprazole and the MR 4‐desmethylpantoprazole‐sulfate/pantoprazole. The correlations were the strongest between 4 and 8 h after pantoprazole administration (spearman ρ ≥ 0.8), suggesting that pantoprazole MR from single plasma samples can be used as a phenotyping metric in patients.

By comparing the effect of the CYP2C19 genotype on AUC_0‐8h_ MR of omeprazole and pantoprazole we demonstrate that both were equivalent in discriminating between different genotype‐predicted phenotypes.

In our analysis, we observed two outlying CYP2C19 IMs with AUC_0‐8h_ MR 5‐hydroxyomeprazole/omeprazole corresponding well with typical values observed for CYP2C19 PMs.^43^ These findings may be explained by rare PM variants that were not identified during genotyping and subsequently wrongly assigned IM status or unexplained phenotypic variability. Nonetheless, our results were still robust after exclusion of these two IMs from the correlation analysis.

Pantoprazole could be a promising candidate for CYP2C19 phenotyping from the long‐term medication, because it has a major advantage over the validated CYP2C19 probe drug omeprazole due to its less pronounced inhibitory effect on CYP2C19.^18^, ^22^, ^24^ Especially in multi‐medicated older adults, the intake of PPIs is highly prevalent,^44^ and these patients, in particular, may profit from CYP2C19 phenotyping due to an increased risk of CYP2C19 phenoconversion. Among PPIs, pantoprazole is the most prescribed in Germany,^45^ and it is one of the most commonly used PPIs worldwide.^46^, ^47^ Due to its high prevalence of use, lack of relevant CYP2C19 inhibition and the lack of an appropriate CYP2C19 biomarker, pantoprazole is a promising candidate for CYP2C19 phenotyping from the prescribed medication in a polypharmacy setting. In a setting of polypharmacy with vulnerable patients, the proposed approach has advantages to previous attempts using a ^13^C pantoprazole breath test for CYP2C19 activity assessment,^27^, ^28^, ^29^ because using pantoprazole MR from the already prescribed medication does not necessitate its previous administration, and it has no additional risk of adverse drug reactions (ADR). Although, a ^13^C pantoprazole breath test is less invasive with regard to sampling, an additional administration of 100 mg ^13^C pantoprazole,^29^ may potentially lead to ADR and drug–drug interactions.

In our study, we used a single‐dose administration of pantoprazole and were able to identify an ideal time range of 4 to 8 h after administration for MR measurements, aiming to phenotype CYP2C19 activity. Although, we did not test the applicability of pantoprazole MR for CYP2C19 phenotyping from multiple dosing, literature suggests that the results could be transferred to a multiple dosing scenario, because the plasma half‐life of pantoprazole and its metabolite 4‐desmethylpantoprazole‐sulfate is relatively short with around 1 and 1.5 h, respectively.^48^ With the intake of a single dose of pantoprazole per day, no accumulation with multiple dosing is to be expected due to its fast elimination.^20^, ^24^, ^49^ Usual doses of pantoprazole are 20 and 40 mg once daily,^50^ due to a dose linearity from 20 to 120 mg of oral pantoprazole,^51^ and the use of MRs to correct for exposure, we expect that CYP2C19 activity of patients taking in different doses of up to 120 mg pantoprazole may be reliably assessed. Further studies with patients using pantoprazole as long‐term medication, preferably in a polypharmacy setting, are warranted to assess the ability of the pantoprazole MR to detect phenoconversion, e.g. by drug–drug interaction.

To be able to form MRs and due to the high instability of the CYP2C19 metabolite 4‐desmethylpantoprazole we chose to assess the subsequent phase II metabolite 4‐desmethylpantoprazole‐sulfate generated by sulfotransferases.^42^ The analytical standard 4‐desmethylpantoprazole‐sulfate and the internal standard 4‐desmethylpantoprazole‐sulfate‐d3 were synthesized using a novel synthesis route for O‐sulphates from 4‐methoxypyridine and 2‐methoxypyridine residues. The standards were used to develop and validate a fast LC–MS/MS method for 4‐desmethylpantoprazole‐sulfate and pantoprazole in human plasma. To our knowledge, this is the first time, an LC–MS/MS method for the quantification of 4‐desmethylpantoprazole‐sulfate in human plasma is described. Only single studies have mentioned or measured 4‐desmethylpantoprazole‐sulfate.^28^

A limitation of this work is, that due to 4‐desmethylpantoprazole‐sulfate being a phase II metabolite, it cannot be fully ruled out that variability in sulfotransferase (SULT) activity may influence the MR 4‐desmethylpantoprazole‐sulfate/pantoprazole.^52^ In this study, however, we were able to demonstrate strong correlations between omeprazole MR and pantoprazole MR that were consistently stronger than using the pantoprazole AUC_0‐8h_ or single plasma concentrations of pantoprazole alone. This clearly supports the notion, that the initial CYP2C19‐mediated formation of 4‐desmethylpantoprazole is the rate‐limiting step in 4‐desmethylpantoprazole‐sulfate formation in humans and that variability in SULT activity may play a lesser role. The future identification of the specific SULT isoform(s) responsible for this metabolic step may allow us to estimate their influence more precisely. There were no genotype‐predicted CYP2C19 PM and UM identified within the study cohort, and we did not assess the response of pantoprazole MRs to CYP2C19 inhibitors and inducers. Further and larger studies are needed to address these important aspects.

In conclusion, we propose the new MR of 4‐desmethylpantoprazole‐sulfate/pantoprazole as a promising CYP2C19 phenotyping approach that showed comparable results to the established probe drug omeprazole in a single‐dose pharmacokinetic study. In addition, we describe a novel synthesis route for 4‐desmethylpantoprazole‐sulfate to support the measurement of pantoprazole MR. Pantoprazole may be superior to omeprazole MRs as they could be used in situations where pantoprazole is prescribed in vulnerable patients, such as multi‐medicated older adults. Phenotyping by pantoprazole during repeated dosing is likely to be more reliable and accurate than omeprazole, due to its reproducible pharmacokinetics without accumulation and the lack of relevant CYP2C19 inhibition.

## AUTHOR CONTRIBUTIONS

J.P.M. and K.S.J. wrote the manuscript, J.P.M., S.Y., B.M., M.H., V.N.B., J.T., T.L. and C.B. performed the research, J.P.M., J.C.S. and K.S.J. designed the research, J.P.M. and S.Y. analysed the data, V.N.B. contributed new reagents/analytical tools.

## CONFLICT OF INTEREST STATEMENT

The authors declared no competing interests for this work.

## Supporting information


**Figure S1.** Synthesis of 4‐desmethylpantoprazole‐4‐O‐sulfate (as triethylammonium salt).
**Figure S2.** Preparation of isotopically labelled analogue starting compound **2**‐D used in the synthesis of 4‐desmethylpantoprazole‐4‐O‐sulfate (**9**‐D) with 3‐(trideuteriomethoxy) group attached to C‐3 in the pyridine ring. For “steps”, see text below.
**Figure S3:** LC–MS fragmentation spectra of A: 4‐desmethylpantoprazole‐sulfate and B: 4‐desmethylpantoprazole‐sulfate‐d3. Mass spectra were generated in negative ion electrospray ionization mode with a collision energy of ‐30 V.
**Figure S4:** H1‐NMR spectrum of 4‐desmethylpantoprazole‐sulfate (triethylammonium salt).
**Figure S5:** H1‐NMR spectrum of 4‐desmethylpantoprazole‐sulfate‐d3 (triethylammonium salt).
**Figure S6:** Retention times of pantoprazole and 4‐desmethylpantoprazole‐sulfate in the novel LC–MS method.
**Figure S7:** Chromatographic traces of 4‐desmethylpantoprazole‐sulfate and the internal standard 4‐desmethylpantoprazole‐sulfate in A: plasma sample of a study participant after oral intake of 20 mg pantoprazole and B: calibrator sample prepared in bovine serum.
**Figure S8:** Rapid degradation of the 4‐desmethylpantoprazole standard in a mixture of methanol and water 40/60% (v/v) at 10 °C. 4‐desmethylpantoprazole was measured with a LC–MS/MS method with a MRM transition in positive ion mode with *m/z* 370 ➔ 186. The graph represents multiple injections of a single sample. The half‐life of 4‐desmethylpantoprazole decay was assessed using GraphPad Prism 10 One Phase Exponential Decay function and resulted in a value of 67.5 min.
**Figure S9.** Schematic depiction of CYP2C19‐dependent pantoprazole metabolism in humans. The molecular structures of pantoprazole, 4‐desmethylpantoprazole and 4‐desmethylpantoprazole‐sulfate are given with the tautomeric shift of 4‐desmethylpantoprazole. The free hydroxyl‐group at the C‐4 together with a neighbouring methoxy‐group (donor substituent) at the C‐3 position of the pyridine ring is prone to oxidation. The sulphate‐conjugation improves stability.
**Figure S10:** Spearman correlation of AUC_0‐8h_ MR omeprazole to AUC_0‐8h_ MR pantoprazole and the pantoprazole AUC_0‐8h_ shown as non‐ln transformed data. A: Correlation of the AUC_0‐8h_ MR 5‐hydroxyomeprazole/omeprazole to AUC_0‐8h_ MR 4‐desmethylpantoprazole‐sulfate/pantoprazole. B: Correlation of AUC_0‐8h_ MR 5‐hydroxyomeprazole/omeprazole to AUC_0‐8h_ pantoprazole. C: Correlation of the AUC_0‐8h_ MR 5‐hydroxyomeprazole/omeprazole to AUC_0‐8h_ MR 4‐desmethylpantoprazole‐sulfate/pantoprazole after exclusion of two outlying IM subjects. D: Correlation of AUC_0‐8h_ MR 5‐hydroxyomeprazole/omeprazole to AUC_0‐8h_ pantoprazole after exclusion of two outlying IM subjects. Spearman ρ is given and a p‐value < 0.05 was considered significant. AUC: area under the curve; MR: metabolic ratio; IM: intermediate metabolizer; NM: normal metabolizer; RM: rapid metabolizer.
**Table S1:** Average accuracy (%) and precision (CV in %) of omeprazole and 5‐hydroxyomeprazole calibrators and quality control samples in bovine serum over all measured study sample batches.
**Table S2:** Average accuracy (%) and precision (CV in %) of pantoprazole and 4‐desmethylpantoprazole‐sulfate calibrators and quality control samples in bovine serum over all measured study sample batches.
**Table S3:** Intraday and interday accuracy and precision for pantoprazole and 4‐desmethylpantoprazole‐sulfate in quality control samples in bovine serum. For interday accuracy and precision a set of calibrators and quality controls were prepared and measured separately on three different days. For intraday accuracy and precision 5 sets of quality control samples and a set of calibrators were prepared and measured on a single day.
**Table S4:** Stability assessments of pantoprazole and 4‐desmethylpantoprazole‐sulfate in bovine serum. For each nominal concentration level n = 3 quality control samples were stored for the indicated conditions and were measured against a freshly prepared set of calibrators and quality control samples. The average accuracy (%) and CV (%) of the quality control samples after 24 h at 10 °C in the LC–MS autosampler after sample preparation (Autosampler stability), for 24 h at room‐temperature before sample preparation (Benchtop stability), and after three freeze–thaw cycles (3x freeze–thaw stability) are shown.
**Table S5:** Assessment of matrix effect using the matrix factor. Pantoprazole and 4‐desmethylpantoprazole‐sulfate were spiked into readily prepared matrices at two concentration levels (low and high). The matrix factor was calculated using the respective internal standards. Internal standard corrected matrix factors were compared to matrix factors without the sample matrix. The coefficient of variation (%) is shown. A CV% below 15% was interpreted as acceptable. n = 5 for low QC and n = 6 for high QC.
**Table S6:** Assessment of dilution Integrity was performed by spiking bovine serum with pantoprazole and 4‐desmethylpantoprazole‐sulfate to a final concentration of 4800 and 3200 ng ml^−1^, respectively. Prior to sample preparation, samples were further diluted with bovine serum with a dilution factor of 1/5 and 1/10. N = 3 samples of each dilution factor were measured against a calibration curve. Average accuracy (%) and CV (%) are given.
**Table S7:** descriptive statistics of the study participants.
**Table S8:** CYP2C19 genotype of the participants.

## Data Availability

The data are available from the corresponding author upon reasonable request.
