# Supplementary material for: Evaluation of a pantoprazole and 4‐desmethylpantoprazole‐sulfate metabolic ratio as a novel CYP2C19 phenotyping method
Source: Br J Clin Pharmacol. 2026 May 31;92(10):3387–96. doi: 10.1002/bcp.70631 (PMC13619035; doi:10.1002/bcp.70631)
Supplement: Supplementary file 1 [file BCP-92-3387-s001.docx]

**Supplemental information to:**

**Evaluation of a pantoprazole and 4-desmethylpantoprazole-sulfate metabolic ratio as a novel CYP2C19 phenotyping method**

Julian Peter Müller^1^, Vladimir N Belov^2^, Sabrina Yamoune^1^, Bob Miller^1^, Mohammed Hasoumi^1^, Jolanta Tupiec^1^, Thea Laurentius^3,4^, L Cornelius Bollheimer^3^, Julia C Stingl^1,5^ and Katja S Just^1^

1 Institute of Clinical Pharmacology, University Hospital RWTH Aachen, Aachen, North Rhine-Westphalia, Germany

2 Department of NanoBiophotonics, Max Planck Institute for Multidisciplinary Sciences (MPI-NAT), Göttingen, Lower Saxony, Germany.

3 Department of Geriatric Medicine, University Hospital RWTH Aachen, Aachen, North Rhine-Westphalia, Germany

4 Department of Geriatrics, Carl von Ossietzky University of Oldenburg, Oldenburg, Lower Saxony, Germany

5 Department of Clinical Pharmacology and Pharmacoepidemiology, University Hospital Heidelberg, Heidelberg, Baden-Württemberg, Germany

**Corresponding author:**

Professor Dr. med. Katja S. Just
Institute of Clinical Pharmacology

University Hospital RWTH Aachen

Wendlingweg 2

D-52074 Aachen

Germany

email: kjust@ukaachen.de

**Synthesis of 4-desmethylpantoprazole-4-O-sulfate and its trideuteriomethoxy analog**

The target structures were 4-desmethylpantoprazole-4-O-sulfate (**9**-H, Figure S1) ^1^ and its labeled analog with trideuteriomethoxy group attached to C-3 in the pyridine ring (**9**-D, Figure S2). Though selective demethylation at C-4 in the pyridine ring of pantoprazole is possible, the product of this transformation cannot be sulfated selectively at the liberated oxygen atom at C-4 of the pyridine ring, without involving other nucleophilic centers of the molecule. Sulfation requires the use of sulfur trioxide or its complexes. Apart from oxygen at C-4 of the pyridine ring, SO_3_ will attack and bind with other nucleophilic centers of 4-desmethylpantoprazole: imidazole nitrogen and oxygen in sulfoxide. Protection of the sulfoxide oxygen is practically impossible. Thus, the synthesis of metabolite **9**-H from pantoprazole was considered to be non-feasible, and another route had to be worked-out (see Figure S1). 2-Chloromethyl-3,4-dimethoxypyridine (**2**-H) and its labelled counterpart **2**-D (Figure S2) were required as starting materials for the preparation of natural **9**-H metabolite and the labelled analog **9**-D. Compound **2**-H was obtained directly from commercially available alcohol **1** in one step (Figure S1). The synthesis of **2**-D (Figure S2) was performed via multiple (trivial) steps. The key intermediate **4** – pantoprazole molecule with non-oxidized S-atom - was prepared from commercially available mercaptoimidazole **3** which was S-alkylated by chloride **2**-H according to the published procedure ^2^. Selective demethylation at C-4 leading to compound **5** (presented in Figure S1 as 4-hydroxypyridine tautomer) was performed by heating of compound **4** with anhydrous sodium sulfide in N-methylpyrrolidone, as described in the previous reference. For the protection of imidazole nitrogens in compound **5**, we used 2,2,2-trichloethyl)oxycarbonyl (Troc) group for several reasons. Initially (under basic conditions), Troc chloride is unselective and reacts with NH site in imidazole nitrogen *and* oxygen atom at C-4 of the pyridine ring. But at the second step, when the intermediate with two Troc residues is treated with strong acid and water, the Troc group is cleaved-off from the oxygen atom at C-4, so that the overall transformation **5** 🡪 **6a** + **6b** (Figure S1) occurs. Isomers **6a** and **6b** were isolated in good overall yield of 61% (calculated over two steps). Importantly, N-Troc group was stable under sulfation conditions, under which isomers **6a,b** were treated with SO_3_ – pyridine complex in DMF – pyridine solution (for an example, see the work by Ridley and colleagues ^3^. This procedure afforded the mixture of *O*-sulfates **7a,b** with Troc-protected imidazoline nitrogen. Thirdly, the electron-acceptor character of 2,2,2-trichloethyl residue makes the Troc group attached to imidazole susceptible to hydrolysis at pH = 9, while *O*-sulfate remains stable under these conditions. The course of these reactions (sulfation and *N*-deprotection) was monitored by means of HPLC. When conversion of **6a,b** to **7a,b** was complete, the solvents (DMF and pyridine) were removed in vacuo, and the residue was carefully introduced into the ice-cold 1 M aqueous triethylamine bicarbonate buffer (pH 9). The product – *O*-sulfate **8** – has much shorter retention time in HPLC than compounds **7a,b**. Upon complete conversion of **7a,b** to **8**, the reaction mixture (pH control: pH>5) was lyophilized, and compound **8** isolated as triethylammonium salt by means of preparative HPLC with aqueous triethyl amine – bicarbonate buffer (pH = 8) and acetonitrile. Oxidation of compound **8**-H into the final product **9**-H was performed at 0°C by careful addition of 12% aq. NaOCl (c = 1.7 M; in portions of 1 equiv.) and water to the vigorously stirred solution of **8**-H in acetonitrile followed by HPLC analysis ^4^. The retention time of sulfoxides **9**-H,D is somewhat shorter than the retention time of **8**-H,D. The reaction was complete upon addition of 2 – 4 equiv. NaOCl. Excess of NaOCl was destroyed by addition of aq. NaHSO_3_, the reaction mixture lyophilized, and the products **9**-H,D isolated by means of preparative HPLC.

The same synthetic methodology, as given in Figure S1, was used for the preparation of isotopically labelled analog (**9**-D) with 3-(trideuteriomethoxy) group (Figure S2).

Figure S1. Synthesis of 4-desmethylpantoprazole-4-O-sulfate (as triethylammonium salt).

Figure S2. Preparation of isotopically labelled analog starting compound **2**-D used in the synthesis of 4-desmethylpantoprazole-4-O-sulfate (**9**-D) with 3-(trideuteriomethoxy) group attached to C-3 in the pyridine ring. For “steps”, see text below.

The transformation of compound **10** to compound **11** and then to chloride **2**-D – the analog of compound **2**-H – are trivial and were performed according to the published procedures involving unlabeled analogs. No D-H exchange was observed. First, 3-hydroxy-2-methyl-4*H*-pyran-4-one (maltol **10**) was *O*-methylated at C-3 with CD_3_I (isotopic purity >98% atom.) according to the known method ^5^. The product - 3-([D_3_]-methoxy)-2-methyl-4*H*-pyran-4-one (**11**) – was converted into 3-([D_3_]-methoxy)-2-methyl-4(1*H*)-pyridone by heating with conc. aqueous NH_3_ ^6^. The following steps leading to compound **2**-D are not shown in Scheme 2. 3-([D_3_]-methoxy)-2-methyl-4(1*H*)-pyridone was transformed into 4-chloro-3-([D_3_]-methoxy)-2-methylpyridine by heating at 90 °C in neat POCl_3_ ^7^. This compound was heated at 90°C with 30% aq. H_2_O_2_ in acetic acid, and thus 4-chloro-3-([D_3_]-methoxy)-2-methylpyridine *N*-oxide prepared [see prev. ref.] The following transformations were performed according to a patent by Kohl and colleagues ^8^. 4-Chloro-3-([D_3_]-methoxy)-2-methylpyridine *N*-oxide was introduced into aromatic nucleophilic substitution of activated chlorine atom at C-4. For that, *N*-oxide and sodium methoxide were heated in methanol and thus transformed into 3-([D_3_]-methoxy)-4-methoxy-2-methylpyridine *N*-oxide. The latter was dissolved in acetic anhydride and heated at 90°C for several hours. In this step, *O*-acetylation occurs at the *N*-oxide site followed by cleavage of N-O bond and 1,3-shift of the acetoxy group to the neighboring carbon at C-2. The product is 2-(acetoxymethyl)-3-([D_3_]-methoxy)-4-methoxy-pyridine. It is hydrolyzed by stirring at 80 °C with 2 M aq. NaOH, and thus 3-([D_3_]-methoxy)-4-methoxy-2-(hydroxymethyl)pyridine synthesized. The latter is transformed into 2-(chloromethyl)-3-([D_3_]-methoxy)-4-methoxy-pyridine (**2**-D in Scheme 2) by dissolution in dichloromethane, addition of thionyl chloride and keeping for several hours at room temperature.

1	Huber, R., Hartmann, M., Bliesath, H., Lühmann, R., Steinijans, V. W. & Zech, K. Pharmacokinetics of pantoprazole in man. *International journal of clinical pharmacology and therapeutics* **34**, 185-194 (1996).

2	Gurjar, M. K., Tripathy, N.K., Maikap, G.S., Mahale, R.D., Khaladkar, T.P., Chaudhari, A.T., Pawar, S.S., Kalhapure, V.K., Mehta, S.S., inventors; Emcure Pharmaceuticals Limited, Bhosari (IN), assignee. Pyridone Derivatives As Acid Secretion Inhibitors And Process For Preparation Thereof. Patent No. US20150232467A1. August 20, 2015

3	Ridley, C. P., Reddy, M. V., Rocha, G., Bushman, F. D. & Faulkner, D. J. Total synthesis and evaluation of lamellarin alpha 20-Sulfate analogues. *Bioorganic & medicinal chemistry* **10**, 3285-3290, doi:10.1016/s0968-0896(02)00237-7 (2002).

4	Rajnikant Vyas, J., Mallik Yadav, K., inventors; Dishman Pharmaceuticals and Chemicals Ltd., assignee. A Preparation Method For Substituted 2-(2-Pyridilmethoylsulphinyl)-1-H-Benzimid Azoles. Patent No. WO2006/049486 A1. May 11, 2006

5	Fisher, B. E. & Hodge, J. E. The Structure of Isomaltol. *The Journal of Organic Chemistry* **29**, 776-781, doi:10.1021/jo01027a002 (1964).

6	Hwang, D. R., Proctor, G. R. & Driscoll, J. S. Pyridones as Potential Antitumor Agents II: 4-Pyridones and Bioisosteres of 3-Acetoxy-2-pyridone. *Journal of Pharmaceutical Sciences* **69**, 1074-1076, doi:<https://doi.org/10.1002/jps.2600690923> (1980).

7	Kohl, B., Sturm, E., Senn-Bilfinger, J., Simon, W. A., Krüger, U., Schaefer, H., Rainer, G., Figala, V. & Klemm, K. (H+,K+)-ATPase inhibiting 2-[(2-pyridylmethyl)sulfinyl]benzimidazoles. 4. A novel series of dimethoxypyridyl-substituted inhibitors with enhanced selectivity. The selection of pantoprazole as a clinical candidate. *Journal of medicinal chemistry* **35**, 1049-1057, doi:10.1021/jm00084a010 (1992).

8	Kohl, B., Muller, B., Haag, D., Simon, W-A., Zech, K., David, M., Von Richter, O., Huth, F., inventors; ALTANA Pharma AG, assignee. Isotopically Substituted Proton Pump Inhibitors. Patent No. WO 2007/012650 A1. February 1, 2007

Figure S3: LC-MS fragmentation spectra of A: 4-desmethylpantoprazole-sulfate and B: 4-desmethylpantoprazole-sulfate-d3. Mass spectra were generated in negative ion electrospray ionization mode with a collision energy of -30V.

Figure S4: H1-NMR spectrum of 4-desmethylpantoprazole-sulfate (triethylammonium salt).

Figure S5: H1-NMR spectrum of 4-desmethylpantoprazole-sulfate-d3 (triethylammonium salt).

Figure S6: Retention times of pantoprazole and 4-desmethylpantoprazole-sulfate in the novel LC-MS method.

Figure S7: Chromatographic traces of 4-desmethylpantoprazole-sulfate and the internal standard 4-desmethylpantoprazole-sulfate in A: plasma sample of a study participant after oral intake of 20 mg pantoprazole and B: calibrator sample prepared in bovine serum.

Table S1: Average accuracy (%) and precision (CV in %) of omeprazole and 5-hydroxyomeprazole calibrators and quality control samples in bovine serum over all measured study sample batches.

|  | Nominal concentration (ng ml^-1^) | CV (%) | Averace accuracy (%) |
| --- | --- | --- | --- |
| Omeprazole calibration | 0.25 | 10.04 | 97.15 |
|  | 0.5 | 8.01 | 106.18 |
|  | 2 | 6.77 | 96.32 |
|  | 10 | 7.35 | 109.93 |
|  | 100 | 4.55 | 92.69 |
|  | 200 | 3.28 | 91.42 |
|  | 800 | 6.30 | 106.33 |
| Omeprazole QC | 0.25 | 11.40 | 132.16 |
|  | 0.5 | 6.48 | 105.62 |
|  | 60 | 6.69 | 119.09 |
|  | 150 | 6.91 | 106.02 |
|  | 600 | 7.00 | 121.64 |
| 5-OH-omeprazole calibration | 0.25 | 18.11 | 98.60 |
|  | 0.5 | 7.17 | 103.81 |
|  | 2 | 7.91 | 94.22 |
|  | 10 | 5.54 | 109.39 |
|  | 100 | 5.13 | 96.18 |
|  | 200 | 5.21 | 97.83 |
| 5-OH-omeprazole QC | 0.25 | 28.02 | 85.59 |
|  | 0.5 | 13.76 | 93.92 |
|  | 60 | 14.53 | 111.33 |
|  | 150 | 7.02 | 99.34 |

CV: coefficient of variation, QC: quality control

Table S2: Average accuracy (%) and precision (CV in %) of pantoprazole and 4-desmethylpantoprazole-sulfate calibrators and quality control samples in bovine serum over all measured study sample batches.

|  | Nominal concentration (ng ml^-1^) | CV (%) | Average accuracy (%) |
| --- | --- | --- | --- |
| Pantoprazole calibration | 3 | 3.86 | 95.42 |
|  | 6 | 3.34 | 107.74 |
|  | 15 | 6.43 | 101.89 |
|  | 75 | 6.35 | 106.73 |
|  | 150 | 3.09 | 104.80 |
|  | 600 | 4.95 | 99.23 |
|  | 1500 | 5.48 | 93.95 |
|  | 2400 | 3.96 | 90.25 |
| Pantoprazole QC | 9 | 5.71 | 107.03 |
|  | 27 | 4.64 | 113.51 |
|  | 450 | 3.48 | 108.00 |
|  | 1350 | 4.31 | 100.22 |
|  | 2160 | 3.23 | 96.01 |
| Pantoprazole-sulfate calibration | 4 | 9.08 | 98.65 |
|  | 10 | 6.64 | 101.31 |
|  | 50 | 7.03 | 107.51 |
|  | 100 | 4.92 | 106.61 |
|  | 400 | 4.07 | 101.90 |
|  | 1000 | 6.69 | 95.36 |
|  | 1600 | 4.36 | 88.67 |
| Pantoprazole-sulfate QC | 12 | 6.33 | 99.70 |
|  | 200 | 4.82 | 100.23 |
|  | 600 | 7.15 | 91.59 |
|  | 960 | 6.71 | 88.12 |
|  | 1440 | 7.68 | 88.80 |

CV: coefficient of variation, QC: quality control, pantoprazole-sulfate: 4-desmethylpantoprazole-sulfate

Table S3: Intraday and interday accuracy and precision for pantoprazole and 4-desmethylpantoprazole-sulfate in quality control samples in bovine serum. For interday accuracy and precision a set of calibrators and quality controls were prepared and measured separately on three different days. For intraday accuracy and precision 5 sets of quality control samples and a set of calibrators were prepared and measured on a single day.

|  | Nominal concentration (ng ml^-1^) | Intraday (n=5) | | Interday (n=3) | |
| --- | --- | --- | --- | --- | --- |
|  |  | CV (%) | Average accuracy (%) | CV (%) | Average accuracy (%) |
| Pantoprazole | 9 | 3.14 | 105.08 | 1.83 | 107.07 |
|  | 27 | 2.43 | 118.97 | 3.31 | 113.15 |
|  | 450 | 3.02 | 114 | 1.99 | 108.26 |
|  | 1350 | 2.97 | 105.72 | 2.02 | 104.02 |
|  | 2160 | 0.89 | 100.05 | 3.18 | 99.03 |
| Pantoprazole-sulfate | 12 | 8.21 | 97.74 | 4.05 | 98.73 |
|  | 200 | 6.16 | 108.03 | 3.83 | 98.64 |
|  | 600 | 3.41 | 96.38 | 5.63 | 94.24 |
|  | 960 | 4.88 | 92.53 | 5.94 | 87.08 |
|  | 1440 | 8.13 | 92.24 | 3.39 | 87.00 |

CV: coefficient of variation, pantoprazole-sulfate: 4-desmethylpantoprazole-sulfate

Table S4: Stability assessments of pantoprazole and 4-desmethylpantoprazole-sulfate in bovine serum. For each nominal concentration level n=3 quality control samples were stored for the indicated conditions and were measured against a freshly prepared set of calibrators and quality control samples. The average accuracy (%) and CV (%) of the quality control samples after 24 h at 10°C in the LC-MS autosampler after sample preparation (Autosampler stability), for 24 hours at room-temperature before sample preparation (Benchtop stability) and after three freeze-thaw cycles (3x freeze-thaw stability) are shown.

|  | Nominal concentration (ng ml^-1^) | Autosampler stability | | Benchtop stability | | 3x freeze-thaw stability | |
| --- | --- | --- | --- | --- | --- | --- | --- |
|  |  | CV (%) | Average accuracy (%) | CV (%) | Average accuracy (%) | CV (%) | Average accuracy (%) |
| Pantoprazole | 9 | 3.28 | 113.15 | 3.57 | 97.79 | 2.1 | 106.66 |
|  | 27 | 2.69 | 121.31 | 0.75 | 113.48 | 2.26 | 113.33 |
|  | 450 | 2.07 | 118.32 | 0.42 | 109.24 | 0.46 | 109.6 |
|  | 1350 | 1.85 | 115.48 | 1.29 | 102.78 | 3.09 | 104.34 |
|  | 2160 | 0.5 | 105.71 | 3.43 | 97.89 | 2.7 | 98.58 |
| Pantoprazole-sulfate | 12 | 1.29 | 102.86 | 5.67 | 95.68 | 3.79 | 95.95 |
|  | 200 | 1.9 | 110.42 | 1.51 | 102.02 | 3.72 | 100.28 |
|  | 600 | 3.78 | 101.11 | 5.76 | 93.41 | 3.2 | 95.23 |
|  | 960 | 4.87 | 94.13 | 1.08 | 85.37 | 1.27 | 86.14 |
|  | 1440 | 3.73 | 93.83 | 3.46 | 85.96 | 4.84 | 89.1 |

CV: coefficient of variation, pantoprazole-sulfate: 4-desmethylpantoprazole-sulfate

Table S5: Assessment of matrix effect using the matrix factor. Pantoprazole and 4-desmethylpantoprazole-sulfate were spiked into readily prepared matrices at two concentration levels (low and high). The matrix factor was calculated using the respective internal standards. Internal standard corrected matrix factors were compared to matrix factors without sample matrix. The coefficient of variation (%) is shown. A CV% below 15% was interpreted as acceptable. n=5 for low QC and n=6 for high QC.

|  | Low QC | | high QC | |
| --- | --- | --- | --- | --- |
|  | CV % | ng/ml | CV % | ng/ml |
| Pantoprazole | 4.22 | 15 | 4.02 | 2160 |
| 4-desmethylpantoprazole-sulfate | 4.01 | 10 | 5.70 | 960 |

QC: quality control

Table S6: Assessment of dilution Integrity was performed by spiking bovine serum with pantoprazole and 4-desmethylpantoprazole-sulfate to a final concentration of 4800 and 3200 ng ml^-1^, respectively. Prior sample preparation, samples were further diluted with bovine serum with a dilution factor of 1/5 and 1/10. N=3 samples of each dilution factor were measured against a calibration curve. Average accuracy (%) and CV (%) are given.

|  | Nominal concentration after dilution (ng ml^-1^) | Dilution factor | Mean (ng ml^-1^) | Backcalculated concentration (ng ml^-1^) | Nominal concentration before dilution (ng ml^-1^) | % CV | Average Accuracy (%) |
| --- | --- | --- | --- | --- | --- | --- | --- |
| Pantoprazole | 480 | 10 | 436.40 | 4364.00 | 4800 | 2.26 | 90.92 |
|  | 960 | 5 | 820.70 | 4103.50 | 4800 | 5.08 | 85.49 |
| 4-desmethylpantoprazole-sulfate | 320 | 10 | 291.20 | 2912.00 | 3200 | 2.03 | 91.01 |
|  | 640 | 5 | 586.30 | 2931.50 | 3200 | 1.94 | 91.61 |

CV: coefficient of variation

Table S7: descriptive statistics of the study participants

| **Parameter** | **n (%)** | **median (range)** |
| --- | --- | --- |
| male | 14 (70) |  |
| female | 6 (30) |  |
| age (years) |  | 24 (22 - 27) |
| BMI (kg/m^2^) |  | 23.5 (19.7 - 28.1) |
| eGFR (ml/min/1.73m^2^) |  | 115.55 (97.6 - 130.2) |

BMI: body mass index, eGFR: estimated glomerular filtration rate

Table S8: CYP2C19 genotype of the participants

| Subject | CYP2C19 genotye | CYP2C19 activity score | Genotype-predicted phenotype |
| --- | --- | --- | --- |
| 1 | *1/*2 | 1 | IM |
| 2 | *1/*2 | 1 | IM |
| 3 | *1/*2 | 1 | IM |
| 4 | *1/*2 | 1 | IM |
| 5 | *1/*1 | 2 | NM |
| 6 | *1/*1 | 2 | NM |
| 7 | *1/*1 | 2 | NM |
| 8 | *1/*1 | 2 | NM |
| 9 | *1/*1 | 2 | NM |
| 10 | *1/*1 | 2 | NM |
| 11 | *1/*1 | 2 | NM |
| 12 | *1/*1 | 2 | NM |
| 13 | *1/*1 | 2 | NM |
| 14 | *1/*1 | 2 | NM |
| 15 | *1/*1 | 2 | NM |
| 16 | *1/*17 | 2.75 | RM |
| 17 | *1/*17 | 2.75 | RM |
| 18 | *1/*17 | 2.75 | RM |
| 19 | *1/*17 | 2.75 | RM |
| 20 | *1/*17 | 2.75 | RM |
|  |  |  |  |

IM: intermediate metabolizer, NM: normal metabolizer, RM: rapid metabolizer

Figure S8: Rapid degradation of the 4-desmethylpantoprazole standard in a mixture of methanol and water 40/60 % (v/v) at 10°C. 4-desmethylpantoprazole was measured with a LC-MS/MS method with a MRM transition in positive ion mode with *m/z* 370 🡪 186. The graph represents multiple injections of a single sample. The half-life of 4-desmethylpantoprazole decay was assessed using GraphPad Prism 10 One Phase Exponential Decay function and resulted in a value of 67.5 minutes.

Figure S9. Schematic depiction of CYP2C19-dependent pantoprazole metabolism in humans. The molecular structures of pantoprazole, 4-desmethylpantoprazole and 4-desmethylpantoprazole-sulfate are given with the tautomeric shift of 4-desmethylpantoprazole. The free hydroxyl-group at the C-4 together with a neighboring methoxy-group (donor substituent) at the C-3 position of the pyridine ring is prone to oxidation. The sulfate-conjugation improves stability.

Figure S10: Spearman correlation of AUC_0-8h_ MR omeprazole to AUC_0-8h_ MR pantoprazole and the pantoprazole AUC_0-8h_ shown as non-ln transformed data. A: Correlation of the AUC_0-8h_ MR 5-hydroxyomeprazole/omeprazole to AUC_0-8h_ MR 4-desmethylpantoprazole-sulfate/pantoprazole. B: Correlation of AUC_0-8h_ MR 5-hydroxyomeprazole/omeprazole to AUC_0-8h_ pantoprazole. C: Correlation of the AUC_0-8h_ MR 5-hydroxyomeprazole/omeprazole to AUC_0-8h_ MR 4-desmethylpantoprazole-sulfate/pantoprazole after exclusion of two outlying IM subjects. D: Correlation of AUC_0-8h_ MR 5-hydroxyomeprazole/omeprazole to AUC_0-8h_ pantoprazole after exclusion of two outlying IM subjects. Spearman ρ is given and a p-value < 0.05 was considered significant. AUC: area under the curve; MR: metabolic ratio; IM: intermediate metabolizer; NM: normal metabolizer; RM: rapid metabolizer
